# Research Hotspots and Frontiers of Transcranial Direct Current Stimulation in Stroke: A Bibliometric Analysis

**DOI:** 10.3390/brainsci13010015

**Published:** 2022-12-21

**Authors:** Chong Li, Shuting Tu, Shuo Xu, Yongli Zhang, Zhijie Yan, Jie Jia, Shiliu Tian

**Affiliations:** 1 School of Exercise and Health, Shanghai University of Sport, Shanghai 200040, China; 2 Institute of Rehabilitation, Fujian University of Traditional Chinese Medicine, Fuzhou 350122, China; 3 Department of Rehabilitation Medicine, Huashan Hospital, Fudan University, Shanghai 200040, China; 4 The Third Affiliated Hospital, Xinxiang Medical University, Xinxiang 453003, China; 5 Key Laboratory of Exercise and Health Science of Ministry of Education, Shanghai University of Sport, Shanghai 200433, China; 6 Shanghai Frontiers Science Research Base of Exercise and Metabolic Health, Shanghai 200031, China; 7 Fujian Sports Vocational Education and Technical College, Fuzhou 350003, China

**Keywords:** tDCS, stroke, CiteSpace, bibliometric, visual analysis

## Abstract

**Background:** Over the past decade, many studies in the field of transcranial direct current stimulation (tDCS) in stroke have been published in scholarly journals. However, a scientometric analysis focusing on tDCS after stroke is still missing. The purpose of this study is to deliver a bibliometric analysis to investigate the global hotspots and frontiers in the domain of tDCS in stroke from 2012 to 2021. **Methods:** Articles and reviews related to tDCS in stroke were retrieved and obtained from the Web of Science core collection database from 2012 to 2021. Data visualization and analysis were conducted by using CiteSpace, VOSviewer, and Microsoft Excel 2019. **Results:** Finally, 371 publications were included in the scientometric analysis, including 288 articles and 83 reviews. The results showed that the number of publications per year increased from 15 to 68 in the last 10 years. Neurosciences was the main research hotspot category (*n* = 201). *Frontiers in Human Neuroscience* was the most published journal with 14 papers. The most productive author, institution, and country were Fregni F (*n* = 13), the League of European Research Universities (*n* = 37), and the United States of America (*n* = 98), respectively. A burstness analysis of keywords and the literature indicated that current studies in the field of tDCS in stroke focused on poststroke aphasia, tDCS combined with robotic therapy, and anatomical parameters. **Conclusion:** The research of tDCS in stroke is predicted to remain a research hotspot in the future. We recommend investigating the curative effect of other different tDCS closed-loop rehabilitation methods for different stroke dysfunctions. In conclusion, this bibliometric study presented the hotspots and trends of tDCS in stroke over the last decade, which may help researchers manage their further studies.

## 1. Introduction

Stroke is a major cause of death and disability globally, with one in four people affected over their lifetime [1]. With the rapid development in medicine, the mortality of strokes is gradually decreasing [2]. For stroke survivors, how to recover function has become their most concerning problem [3]. Studies have found that the excitability asymmetry of bilateral cerebral hemispheres in stroke patients may be one of the causes of dysfunction [4,5,6]. The emergence of noninvasive brain stimulation could effectively help stroke patients restore the balance between the cerebral hemispheres [7,8]. Transcranial direct current stimulation (tDCS) is a noninvasive brain stimulation technique that uses a constant, low-intensity electrical current to modulate neuronal activity in the cerebral cortex [9]. tDCS mainly regulates the resting potential threshold of neurons by applying direct currents to the projection area of the head skin, interferes with the discharge of neurons, and achieves the therapeutic effect of changing the excitability of the cerebral cortex in the stimulated area [10]. Due to its advantages of noninvasiveness, portability, and high safety, tDCS has been widely used by researchers around the world to treat various functional disorders of stroke patients [11,12,13]. In addition, a growing number of papers in recent years have been published in various journals. Nevertheless, a scientometric analysis of studies focused on tDCS in stroke is still lacking.

A bibliometric analysis refers to the multiangle analysis of references, cooperation networks, keywords, etc., of the included studies through metrology software. Based on the pathfinding network algorithm and cocitation analysis theory, CiteSpace [14] and VOSviewer [15] are bibliometric software that can analyze publications of specific disciplines and draw visual atlases to show research hot topics and frontiers. In recent years, bibliometric analyses using CiteSpace and VOS viewer have become a research hotspot for scholars at home and abroad [16,17,18,19,20,21].

To address the lack of quantitative analyses for studies in the domain of tDCS in stroke, a bibliometric analysis was performed using CiteSpace and VOSviewer. The results from this bibliometric study could help researchers quickly understand the research hotspots and frontiers of tDCS in stroke. In addition, our analysis could help investigators plan and manage their scientific work.

## 2. Materials and Methods

### 2.1. Data Source and Search Strategy

Literature data for this bibliometric study were obtained and retrieved from the Web of Science (WoS) core collection database. The terms ‘transcranial direct current stimulation’ and ‘stroke’ were used in the MeSH search, where ‘tDCS’ was represented by other expressions. The search terms were as follows: (TS (transcranial direct current stimulation) OR TS (tDCS)) AND TS (stroke). Time spans were retrieved from 1 January 2012 to 31 December 2021.

### 2.2. Inclusion Criteria

**Figure 1:**
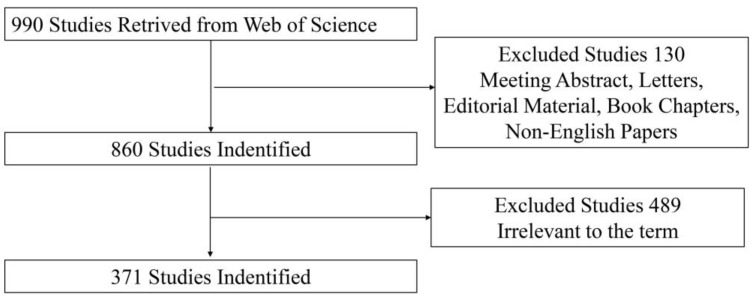
Flowchart of study inclusion criteria.

Articles and reviews in the field of tDCS in stroke were included. The theme was determined according to the title and abstract. We excluded meeting abstracts, letters, news items, and published editorial materials. We restricted the publication language to English. Finally, a total of 371 studies was included (Figure 1).

### 2.3. Data Extraction

CL and STT researched and extracted the publications related to the application of tDCS in stroke from the WoS core collection. A statistical analysis of the downloaded published research data was performed using Microsoft Excel 2019. CiteSpace 5.8.R3 and VOSviewer 1.6.16 were used for the bibliometric analysis of the included studies.

### 2.4. Software Parameter Settings

The LinLog/modularity method was used for the VOSviewer. The parameters of CiteSpace were set as follows: the type of Node was chosen based on the analysis and the ‘Time Sliding’ value was set to 1 year.

## 3. Results

### 3.1. Publication Analysis

**Figure 2:**
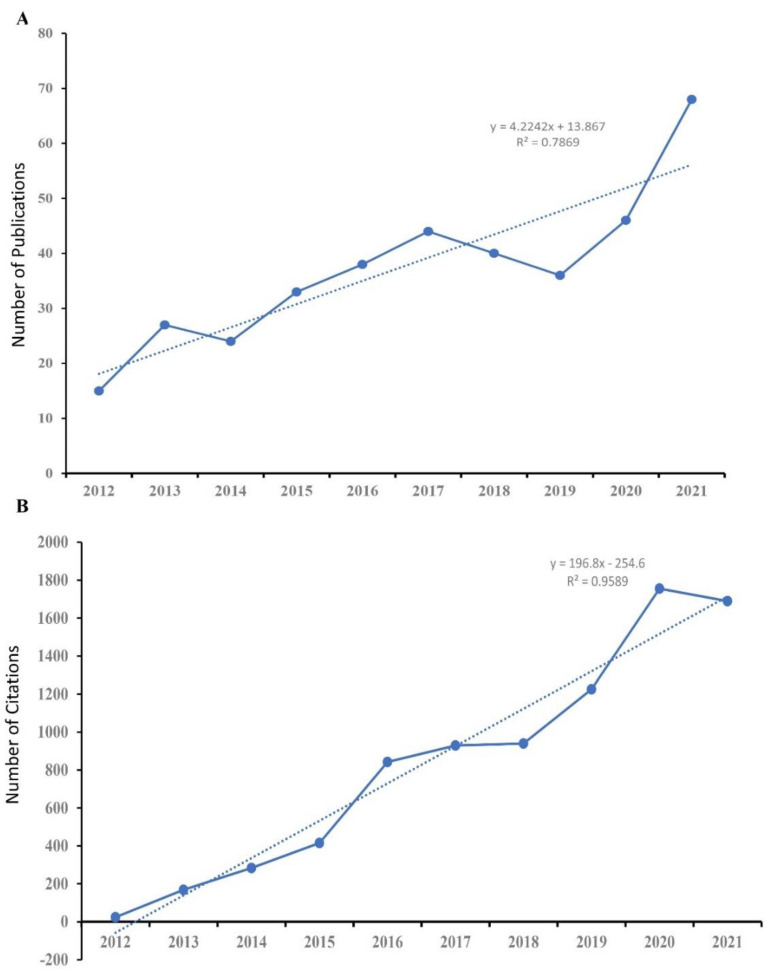
The number of annual publications (**A**) and the sum of annual citations (**B**) in studies of tDCS in stroke from 2012 to 2021.

There were 288 (77.63%) articles and 83 (22.37%) reviews among the 371 publications. The distribution of the annual publication of tDCS in stroke research from 2012 to 2021 is presented in Figure 2A. In 2021, there were 68 annual papers, which was an increase from 15 in 2012. The general trend was upbeat and the time trend of publications over the previous ten years revealed a significant link (R^2^ = 0.7869, *p* < 0.01). From 24 in 2012 to 1690 in 2021, the number of citations per year is shown in Figure 2B. According to a linear regression study, the annual citation trend from 2012 through to 2021 was expected to be positive (R^2^ = 0.9589, *p* < 0.01).

**Figure 3:**
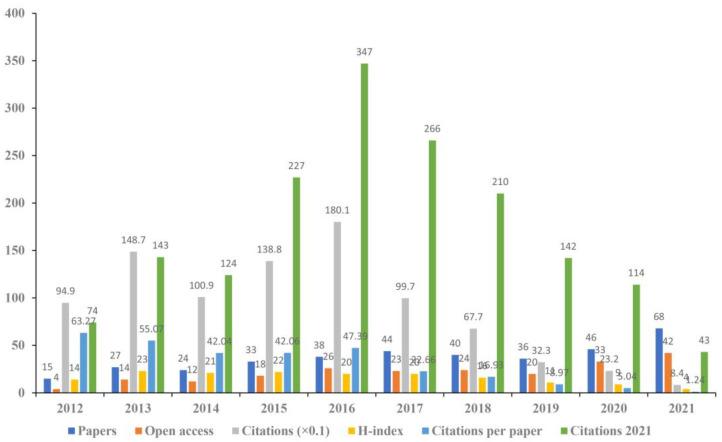
The annual number of papers, open access papers, citations (×0.1), H-index, citations per paper, and citations in 2021 of included studies.

As shown in Figure 3, the highest H-index value was 23 in 2013, the largest number of citations per paper was 63.27 in 2012, the highest number of papers was published in 2021 (68), and the greatest number of open access papers was also observed in 2021 (42). We speculated that the number of publications related to tDCS in stroke was likely to be approximately 90 in 2022, which would suggest that the application of tDCS in stroke would remain a hotspot in the future.

### 3.2. Journal Analysis

The 371 papers included in this study were published in 134 scholarly journals. Information on the top 15 academic journals is summarized in Table 1. The academic journal *Frontiers in Human Neuroscience,* with an impact factor of 3.169 in 2020, published the greatest number of papers (*n* = 28), followed by *Restorative Neurology and Neuroscience* (22 publications, IF = 2.406) and *Frontiers in Neurology* (13 publications, IF = 4.003). In detail, the academic journal *Frontiers in Human Neuroscience* had the highest H-index value (18). *Brain Stimulation* had the largest quantity of citations per paper (84.08). The *Cochrane Database of Systematic Reviews* had the highest IF of 9.289 in 2020. From the perspective of the number of publications and the IF of journals, *Frontiers in Human Neuroscience* might have been the most influential journal.

**Figure 4:**
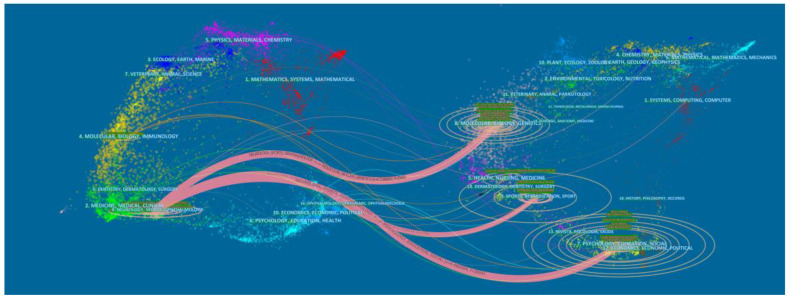
The dual-map overlay of journals in the field of tDCS in stroke.

A dual-map overlay of the journals is shown in Figure 4. The majority of publications was published in ophthalmology, neurology, and sports journals, which were mostly cited in molecular, biology, and genetics journals.

### 3.3. Subject Category Analysis

**Figure 5:**
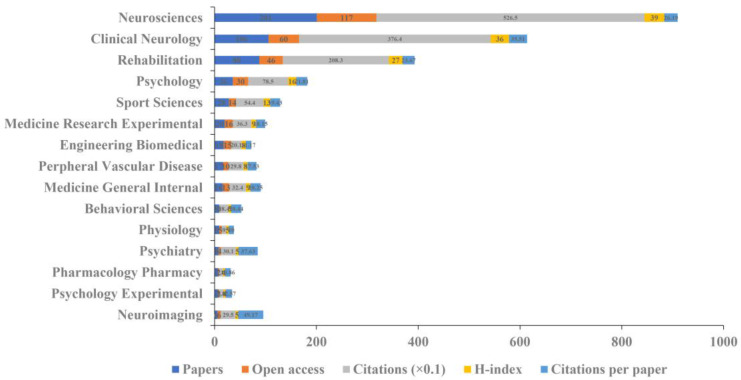
The amounts of papers, open access papers, citations, H-index, and citations per paper of the top 15 subject categories in the WoS.

Information on the top 15 subject categories in the WoS is shown in Figure 5. Specifically, neurosciences had the highest amount of papers (201), citations (5265), open access papers (117), and H-index value (39). Neuroimaging had the largest number of citations per paper (49.17), followed by psychiatry (37.63) and clinical neurology (35.51).

### 3.4. Authoritative Country and Institution Analysis

**Figure 6:**
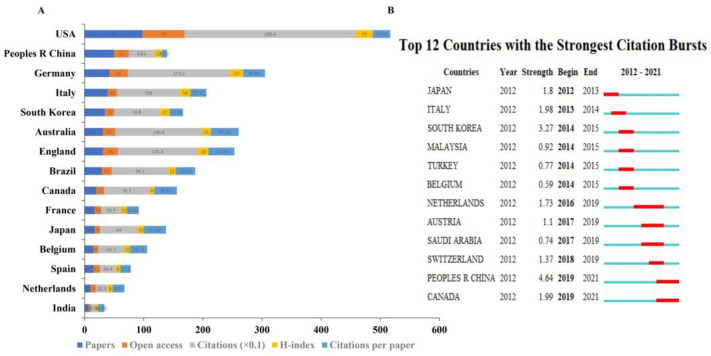
Distribution of global publications. (**A**) The amounts of papers, open access papers, citations, H-index, and citations per paper in the top 15 countries. (**B**) Top 12 countries with the strongest citation bursts using CiteSpace. The blue bars mean the reference was published; the red bars mean citation burstness.

In total, 47 countries were involved in the publication of tDCS in stroke. The top 15 countries based on the number of publications are shown in Figure 6A. The USA had the highest number of publications (98), citations (2894), open access papers (71), and H-index (29). Australia had the greatest number of citations per paper (47.23), followed by England (43.74) and Canada (38.35). The top 12 countries with the strongest citation bursts are shown in Figure 6B. China represented the greatest burst strength, with 4.64 from 2019 to 2021, indicating that many researchers focused on the application of tDCS in stroke in China from 2019 to 2021. In addition, China and Canada had similar burst strengths from 2019 to 2021, which indicated that the latest researches at the time were mainly from those two countries.

**Figure 7:**
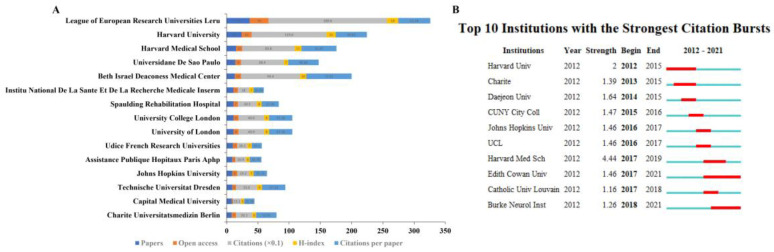
Distribution of global institutions. (**A**) The number of papers, open access papers, citations, H-index, and citations per paper of the top 15 institutions. (**B**) The top 10 institutions according to CiteSpace in terms of citation bursts. The blue bars mean the reference was published; the red bars mean citation burstness.

Information on the top 15 institutions according to the amounts of papers is shown in Figure 7A. The League of European Research Universities (LERU) ranked first in terms of number of publications (37), followed by Harvard University (24) and Harvard Medical School (15). The Beth Israel Deaconess Medical Center had the highest number of citations per paper (72.62). For the burstness analysis of institutions (Figure 7B), the top three institutions were Harvard Medical School from 2017 to 2019, followed by Harvard University from 2012 to 2015 and Charite from 2013 to 2015.

### 3.5. Authoritative Author Analysis

**Figure 8:**
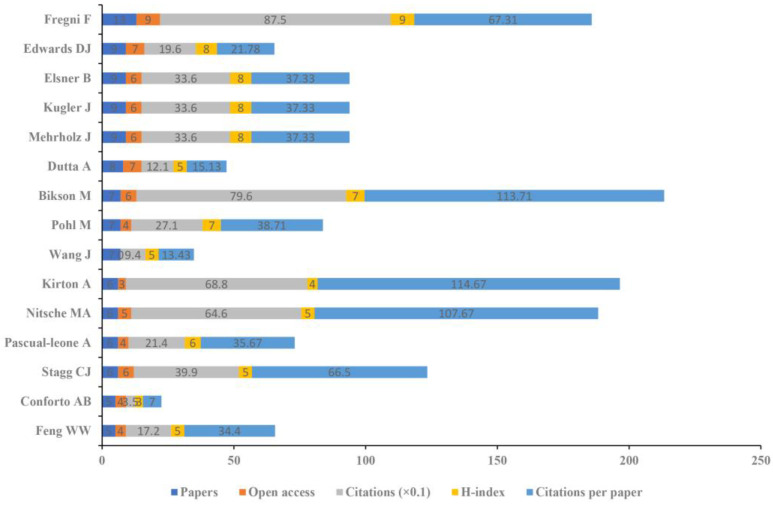
The amounts of papers, open access papers, citations, H-index, and citations per paper of the top 15 authors.

The 371 included studies were published by 1691 authors. The top 15 authoritative authors are summarized in Figure 8. In detail, Fregni F had the largest number of papers (13), open access papers (9), citations (875), and H-index (9). Kirton A had the greatest number of citations per paper (114.67).

### 3.6. Coauthorship Analysis of Countries, Institutions, and Authors

**Figure 9:**
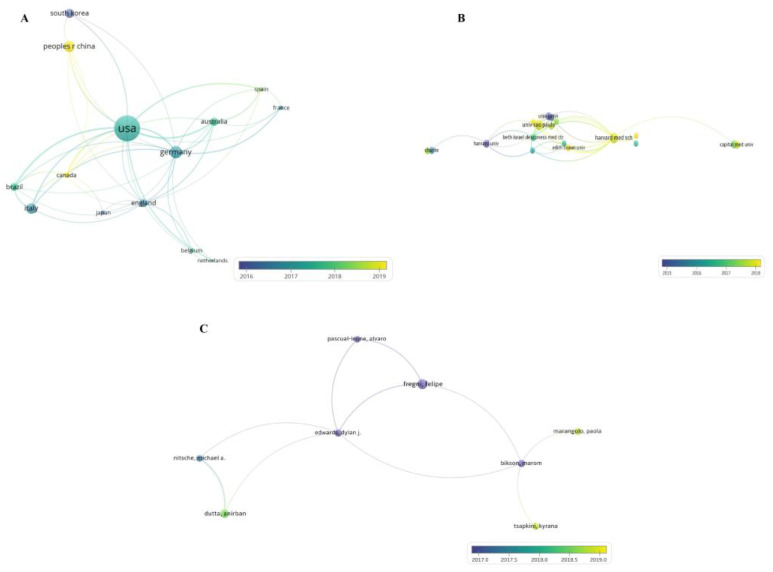
Collaboration maps of coauthorship analysis of countries (**A**), institutions (**B**), and authors (**C**).

The collaboration maps of countries, institutions, and authors are displayed in Figure 9. Based on the total link strength, the top three countries were the UAS (75), Germany (45), and Australia (34) (Figure 9A). In addition, the node in yellow indicated that the average publishing year was 2019. China was the most recent country to publish publications on tDCS in stroke. The top three institutions were the Beth Israel Deaconess Medical Centre (10), Harvard Medical School (10), and University of Sao Paulo (9). The top three authors were Bernhard Elsner (25), Joachim Kugler (25), and Jan Mehrholz (25).

### 3.7. Reference Analysis

**Figure 10:**
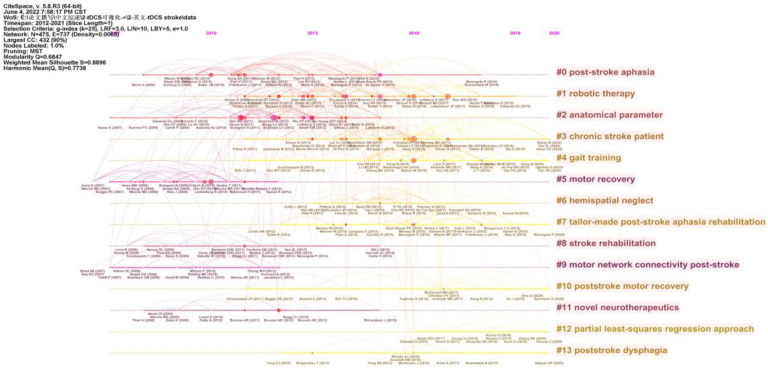
A timeline view of reference cocitation analysis using CiteSpace.

A timeline view of the references is presented in Figure 10. The grouped research categories in the reference cocitation analysis were split into 14 groups (#0–13). With 58 members, the largest cluster (#0) was classified as poststroke aphasia. The most pertinent to the cluster was the “Use of Computational Modeling to Inform tDCS Electrode Montages for the Promotion of Language Recovery in Post-stroke Aphasia” [22]. The second-largest cluster (#1), labeled as robotic therapy, had 51 members. The most relevant citer to the cluster was “Transcranial direct current stimulation (tDCS) for improving activities of daily living, and physical and cognitive functioning, in people after stroke” [23]. The third-largest cluster was labeled as the anatomical parameter. The most relevant citer was “Stroke rehabilitation using noninvasive cortical stimulation: motor deficit” [24].

### 3.8. Keyword Analysis

**Figure 11:**
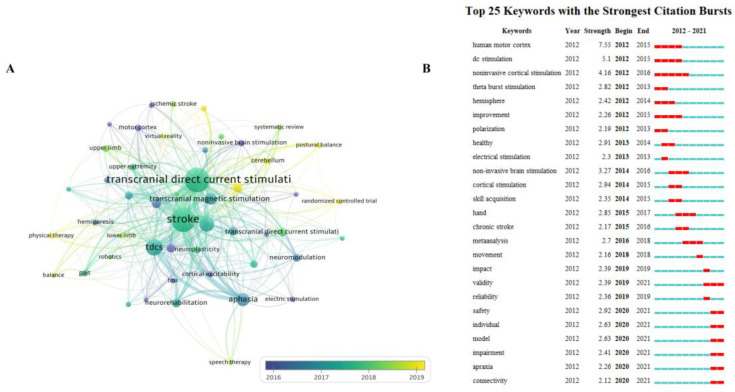
Analysis of author keyword co-occurrence. (**A**). The visual map was obtained using VOSviewer. (**B**). Top 25 keywords with the strongest citation bursts shown using CiteSpace. The blue bars mean the reference was published; the red bars mean citation burstness.

A total of 658 author keywords was retrieved from 371 publications, of which 49 met the threshold. Figure 11A showed the co-occurrence relations of the keywords. Rehabilitation, aphasia, transcranial magnetic stimulation, motor recovery, gait, and dysphagia were high-frequency keywords. The median year of publication for tDCS in stroke was 2017. From 2016 to 2019, the other keywords were used in this field in order. Recently, the combination of tDCS and other therapies (such as physical therapy, virtual reality, TMS, etc.) has attracted the attention of some scholars, indicating that the synergistic therapeutic effect of tDCS is likely to remain a hotspot in future research.

The top 25 keywords with the most powerful citation bursts are displayed in Figure 11B. The keywords with the strongest citation burst since 2012 were human motor cortex, dc stimulation, noninvasive cortical stimulation, theta burst stimulation, hemisphere, improvement, and polarization. The most recent burst keywords were safety, individual, model, impairment, apraxia, and connectivity. The human motor cortex had the highest burst strength of 7.55 from 2012 to 2015.

### 3.9. Analysis of the Top 10 Papers Cited Most Frequently

The top 10 papers cited most frequently are summarized in Table 2. The top 10 papers were cited 1769 times, accounting for 19.77 of the total cited amount (8947). The article by Bikson M [25] with the title “Safety of Transcranial Direct Current Stimulation: Evidence-Based Update 2016”, which was published in 2016 in *Brain Stimulation*, was the most cited article (562 citations), followed by “The contribution of interindividual factors to the variability of response in transcranial direct current stimulation studies” [26] (236 citations) and “Does anodal transcranial direct current stimulation enhance the excitability of the motor cortex and motor function in healthy individuals and subjects with stroke: A systematic review and meta-analysis” [27] (155 citations). The topic of motor recovery was addressed in four of the top ten papers, which may have been a significant factor for the occurrence of the 14 instances of the phrase motor recovery.

## 4. Discussion

### 4.1. Global Research Trends of tDCS in Stroke

tDCS has been applied in stroke rehabilitation for more than a decade [28]. This study presented a bibliometric analysis by using CiteSpace and VOSviewer to measure the studies focused on tDCS in stroke in the last 10 years. The results showed that the global trend of published papers on tDCS in stroke increased over the last 10 years, and was predicted to continue to increase in 2022. In addition, the global trend of citation amounts increased from 24 to 1690, indicating that tDCS has received more attention in the field of stroke in recent years. The majority of included studies was articles, which indicated that more research is being conducted. In terms of the subject category analysis, neurosciences, clinical neurology, and rehabilitation were the main categories that focused on tDCS in stroke. In terms of the journal analysis, the top 15 authoritative journals contributed to 43.66% of the total amount of publications on tDCS in stroke. *Frontiers in Human Neuroscience* had the highest number of papers (28), open access (27), and H-index (16). However, the greatest citation amount (1009) and citations per paper (84.08) occurred in *Brain Stimulation*. Amongst the top 15 journals, only two journals, *Brain Stimulation* (IF, 2020 = 8.955) and *Cochrane Database of Systematic Reviews* (IF, 2020 = 9.289), had IF scores of >5. In summary, it was shown that it is a great challenge to publish papers focused on tDCS in stroke in high-IF journals.

Amongst the top 15 countries based on publication numbers, 13 were developed countries and only 2 (China and India) were developing countries. In terms of research on tDCS in stroke, there was still a significant difference between developed and developing nations. In addition, though the USA had the highest number of citations (2894) and the highest H-index (29), Australia ranked first in terms of citations per paper (47.23). Furthermore, Germany had a greater betweenness centrality (0.67) than the USA (0.39). Therefore, the USA should improve the research quality and strengthen cooperation in this field. Amongst authoritative institutions, the League of European Research Universities (LERU) ranked first in terms of papers (37), open access (30), citations (1896), and H-index (18). Amongst authoritative authors, Fregni F ranked first in terms of publications (13), followed by Edwards DJ and Elsner B.

### 4.2. Research Hotspots of tDCS in Stroke

The evolution of a knowledge domain can be reflected through references. Therefore, a reference analysis can reveal research hotspots. According to the timeline view of the literature analysis, poststroke aphasia, robotic therapy, and anatomical parameter were the top three clusters.

Poststroke aphasia: No evidence of benefits of tDCS (anodal, cathodal, and bihemispheric) versus sham tDCS for improving language impairment in patients with aphasia following stroke was found, according to Cochrane Database Systematic Reviews [29]. However, a meta-analysis [30] and a systematic review [31] indicated that tDCS is effective for stroke aphasia rehabilitation at the chronic stages. In addition, a network meta-analysis [32] showed that anodal tDCS, particularly over the left inferior frontal gyrus, seems to be the most promising tDCS option for improving the performance of naming for stroke patients. Therefore, further studies are needed to investigate the effectiveness of tDCS in the subacute stroke phase. In addition, authors of future research should adhere to the CONSORT statement [33].

Robotic therapy: In recent years, tDCS combined with robotic therapy has been shown to be an innovative rehabilitation approach to improve the function of stroke patients. However, existing studies indicated that there are not enough data about the benefits of tDCS as an add-on intervention to robot-assisted therapy on upper limb function for stroke patients [34]. However, tDCS may enhance the effects of robotic therapy alone for lower limb function [35]. In addition, this study indicated that tDCS parameters and the stage or type of stroke injury could be crucial factors that determine the effectiveness of this therapy [35]. Therefore, randomized controlled studies with large sample sizes are needed in the future to identify the effect of different tDCS parameters combined with robotic therapy for stroke patients.

Anatomical parameter: Anatomical variability in tDCS placement can alter the neuromodulatory effects. Evidence-based guidelines indicated no recommendation for the anodal tDCS of the ipsilesional motor cortex and the cathodal tDCS of the contralesional motor cortex in motor stroke. In addition, no recommendation for the use of anodal tDCS of the left Broca area was seen for nonfluent poststroke aphasia [12]. Future research is required to identify the ideal tDCS target location to enhance poststroke function and the most effective strategy to activate it. Additionally, by focusing on any of the components of the learning network, it may be possible to obtain the same enhancing effect through their interconnectedness [36].

### 4.3. Research Frontiers of tDCS in Stroke

According to the document and author analyses, groups such as the Fregni team, Elsner team, and Vandermeeren team still lead this field. Fregni and his collaborators worked on the safety, efficacy, and acceptability of tDCS in stroke [25,37,38,39,40,41]. In their recent study, they indicated that tDCS is not a useful combination strategy when physical therapy has a large effect by itself [42]. In addition, evidence-based guidelines they proposed recently indicated that tDCS improves motor rehabilitation in chronic and subacute stroke, except when used to enhance robotic therapy [43]. Elsner and his collaborators focused on the effectiveness of tDCS on arm function, cognitive function, and aphasia after stroke. They indicated that there is no evidence of the effectiveness of tDCS (anodal tDCS, cathodal tDCS, and dual tDCS) versus sham tDCS for improving aphasia after stroke (low quality of evidence) [44]. However, in their recent research, they showed that anodal tDCS over the left inferior gyrus seemed to be a promising treatment to improve performance in naming for stroke patients [32]. A review published in the *Cochrane Database Systematic Reviews* journal presented a very low to moderate quality on the effectiveness of tDCS versus control for improving activities of daily living (ADL) after stroke. In addition, evidence of low to high quality indicated that tDCS did not improve upper and lower limb function in stroke patients [45]. Therefore, future studies are needed in this area to foster the base of these findings. Vandermeeren and his colleagues focused on the efficacy of tDCS in stroke motor dysfunction. They found that dual tDCS combined with motor skill learning is promising for poststroke rehabilitation [46,47]. Furthermore, their research indicated that combining tDCS with peripheral nerve stimulation could facilitate motor performance in stroke patients [48]. Our results indicated that many emerging scholars are devoted to tDCS in stroke, but, at present, they lack cooperation with international teams. Future research in this area would benefit from more cooperation and communication.

In recent years, the theory of central–peripheral–central closed-loop rehabilitation has been widely used in stroke rehabilitation [49]. Based on this theory, the closed-loop treatment method formed by tDCS combined with other treatments has also been widely used in stroke rehabilitation. For motor dysfunction after stroke, studies have shown that tDCS combined with physiotherapy, mirror therapy, modified constraint-induced movement therapy, and virtual reality can accelerate the recovery of motor function in patients with stroke [50,51,52,53]. For dysphagia after stroke, studies indicated that tDCS combined with traditional Chinese medicine, dysphagia therapy, and respiratory training could improve dysphagia [54,55,56]. For shoulder pain in stroke, one study indicated that tDCS was not a useful combination strategy when physical therapy had a large effect by itself [42]. At present, different closed-loop tDCS treatment schemes have been gradually applied in stroke rehabilitation. In the future, it is necessary to explore the curative effect of other different tDCS closed-loop rehabilitation methods on different dysfunctions of stroke.

### 4.4. Strengths and Limitations

This was the first study to use CiteSpace and VOSviewer to perform a bibliometric analysis of publications focused on tDCS in stroke in the last 10 years. However, several limitations need to be noted regarding this study. Firstly, we selected publications from 2012 to 2021, but the WoS core collection was updated in 2022, and this part was omitted in the present work. Second, although the terms ‘transcranial direct current stimulation’, or ‘tDCS’, and ‘stroke’, which were in the title, abstract, and keywords, were chosen, technical issues with the program prevented the retrieval and analysis of these terms in the text. Third, we only analyzed data limited to the WoS core collection. Therefore, some documents not included in the WoS core collection were missed.

## 5. Conclusions

This bibliometric study analyzed papers focusing on tDCS in stroke over the past 10 years, and presented new insights for research in this field. The most influential author, institution, journal, and country were Fregni F, the League of European Research Universities, *Frontiers in Human Neuroscience*, and the USA, respectively. Furthermore, the research prospects and hotspots included tDCS for improving different disorders after stroke. Large sample randomized controlled trials are needed to be carried out to determine the effects of different parameters of tDCS on stroke patients in the future. In conclusion, this study could help researchers quickly understand the current hotspots and frontiers in the field of tDCS in stroke.

## Figures and Tables

**Table 1:** The top 15 paper journals based on the number of publications.

Journals	Papers	Citations (WoS)	Citations per Paper	Open Access	WoS Categories	IF (2020)	Quartile	H-Index
*Frontiers in Human Neuroscience*	28	753	26.89	27	NEUROSCIENCES; PSYCHOLOGY	3.169	Q3; Q3	16
*Restorative Neurology and Neuroscience*	22	512	23.27	3	NEUROSCIENCES	2.406	Q4	14
*Frontiers in Neurology*	13	106	8.15	13	CLINICAL NEUROLOGY; NEUROSCIENCES	4.003	Q2; Q2	6
*Journal of Neuroengineering and Rehabilitation*	13	156	12	13	ENGINEERING, BIOMEDICAL; NEUROSCIENCES; REHABILITATION	4.262	Q2; Q2; Q1	5
*Brain Stimulation*	12	1009	84.08	6	CLINICAL NEUROLOGY; NEUROSCIENCES	8.955	Q1; Q1	11
*Neurorehabilitation and Neural Repair*	11	440	40	5	CLINICAL NEUROLOGY; REHABILITATION	3.919	Q2; Q1	10
*Archives of Physical Medicine and Rehabilitation*	8	321	40.13	1	REHABILITATION; SPORT SCIENCES	3.966	Q1; Q2	8
*Neuroscience Letters*	8	161	20.13	1	NEUROSCIENCES	3.046	Q3	5
*Brain Sciences*	7	26	3.71	7	NEUROSCIENCES	3.394	Q3	4
*Clinical Neurophysiology*	7	370	52.86	5	CLINICAL NEUROLOGY; NEUROSCIENCES	3.708	Q2; Q2	6
*Frontiers in Neuroscience*	7	77	11	7	NEUROSCIENCES	4.677	Q2	5
*Journals of Stroke Cerebrovascular Diseases*	7	40	5.71	1	NEUROSCIENCES; PERIPHERAL VASCULAR DISEASE	2.136	Q4; Q4	3
*Trials*	7	46	6.57	7	MEDICINE, RESEARCH & EXPERIMENTAL	2.279	Q4	4
*American Journal of Physical Medicine Rehabilitation*	6	84	14	2	REHABILITATION; SPORTS SCIENCES	2.159	Q3; Q3	5
*Cochrane Database of Systematic Reviews*	6	251	41.83	3	MEDICINE, GENERAL & INTERNAL	9.289	Q1	6

**Table 2:** Top 10 most cited papers in included studies.

Title	First Author	Journal	Impact Factor	Year	Citation (WoS)	WoS Categories	Category Quartile
Safety of Transcranial Direct Current Stimulation: Evidence Based Update 2016	Bikson, M	*BRAIN STIMULATION*	8.955	2016	562	CLINICAL NEUROLOGY; NEUROSCIENCES	Q1; Q1
The contribution of interindividual factors to variability of response in transcranial direct current stimulation studies	Li, LM	*FRONTIERS IN CELLULAR NEUROSCIENCE*	5.505	2015	236	NEUROSCIENCES	Q1
Does anodal transcranial direct current stimulation enhance excitability of the motor cortex and motor function in healthy individuals and subjects with stroke: A systematic review and meta-analysis	Bastani, A	*CLINICAL NEUROPHYSIOLOGY*	3.708	2012	155	CLINICAL NEUROLOGY; NEUROSCIENCES	Q2; Q2
Contralesional Hemisphere Control of the Proximal Paretic Upper Limb following Stroke	Bradnam, LV	*CEREBRAL CORTEX*	5.357	2012	151	NEUROSCIENCES	Q1
Transcranial direct current stimulation (tDCS) and language	Monti, A	*JOURNAL OF NEUROLOGY NEUROSURGERY AND PSYCHIATRY*	10.283	2013	125	CLINICAL NEUROLOGY; PSYCHIATRY; SURGERY	Q1; Q1; Q1
Transcranial direct current stimulation facilitates motor learning post-stroke: a systematic review and meta-analysis	Kang, N	*JOURNAL OF NEUROLOGY NEUROSURGERY AND PSYCHIATRY*	10.283	2016	120	CLINICAL NEUROLOGY; PSYCHIATRY; SURGERY	Q1; Q1; Q1
Predicting behavioural response to TDCS in chronic motor stroke	O’Shea, J	*NEUROIMAGE*	6.556	2014	110	NEUROIMAGING; NEUROSCIENCES; RADIOLOGY, NUCLEAR MEDICINE & MEDICAL IMAGING	Q1; Q1; Q1
Ipsilesional anodal tDCS enhances the functional benefits of rehabilitation in patients after stroke	Allman, C	*SCIENCE TRANSLATIONAL MEDICINE*	17.992	2016	106	CELL BIOLOGY; MEDICINE, RESEARCH & EXPERIMENTAL	Q1; Q1
Effect of Anodal Versus Cathodal Transcranial Direct Current Stimulation on Stroke Rehabilitation: A Pilot Randomized Controlled Trial	Khedr, EM	*NEUROREHABILITATION AND NEURAL REPAIR*	3.919	2013	102	CLINICAL NEUROLOGY; REHABILITATION	Q2; Q1
A meta-analysis of the efficacy of anodal transcranial direct current stimulation for upper limb motor recovery in stroke survivors	Butler, AJ	*JOURNAL OF HAND THERAPY*	1.95	2013	102	ORTHOPEDICS; REHABILITATION; SURGERY	Q3; Q3; Q3

## Data Availability

Data are available on reasonable request.
